# O-34 - UNDERSTAND THE VIEWS OF PEOPLE LIVING IN PRISON ON VACCINATION AND CO-PRODUCING RESOURCES TO PROMOTE UPTAKE: NHS GREATER GLASGOW AND CLYDE EXPERIENCE

**DOI:** 10.1093/pubmed/fdag046.030

**Published:** 2026-07-09

**Authors:** Ms Helen Benson, Jane Beresford, Shona Macleod, Shaun Glowa, Kate Deacon, Briege Nugent

**Affiliations:** Public Health Protection Unit, NHS Greater Glasgow And Clyde; Public Health Protection Unit, NHS Greater Glasgow And Clyde; Public Health Protection Unit, NHS Greater Glasgow And Clyde; Media Education; Media Education; Media Education

## Abstract

**Background:**

Vaccination rates among Scottish prisoners are significantly lower than the general population. Reasons are multifactorial, but include lower engagement and inability to access vaccination information. Evidence shows accessible co-produced resources can promote engagement with health campaigns among marginalised groups.

**Objectives:**

To undertake research to inform creation of co-produced resources to encourage vaccination uptake in GGC prisons.

**Methods:**

Following an evidence review, focus groups with people living in three Glasgow custodial settings were undertaken to gather views on vaccination and preferred methods for receiving health information. These were followed by interviews with prison healthcare and Scottish Prison Service staff.

**Results:**

Focus group participants reported the absence of accessible vaccination information meant they were reliant on peer messaging, often leading to misinformation. They wanted clear messages, delivered by health professionals and including peer voices. Key themes for inclusion were their right to vaccination to protect their health and that of others, promoting awareness of vaccine eligibility and infectious disease risk in prison, and addressing the misconception that vaccines were principally for the very young or older adults. Prison was considered as a time to take care of your health and participants stated the opportunity for vaccination should be part of this.

We co-produced videos, radio clips and posters; for longevity and transferability these are generic rather than based on specific vaccination campaigns. Videos include interviews and voices of peers and prison healthcare staff, with animations.

**Conclusions:**

Our findings highlight the importance of filling information voids in prisons by providing co-produced readily accessible vaccination information to inform decision making. Resources were launched across custodial settings in Glasgow ahead of the 2025 Winter Vaccination Programme. A subsequent pilot of weekly vaccination clinics at Glasgow’s largest prison provided regular opportunities for non-routine and seasonal vaccination, further facilitating uptake.

